# An Immediate‐Response Detection System for γ‐Hydroxybutyrate to Enhance Personal Safety in Social Environments

**DOI:** 10.1002/advs.76399

**Published:** 2026-07-06

**Authors:** Jai Eun An, Jisung Kwak, Kyung Ho Kim, Chaeeun Kim, Sung Eun Seo, Nathaniel S. Hwang, Yong‐Sang Ryu, Hyun Seok Song, Oh Seok Kwon

**Affiliations:** ^1^ Interdisciplinary Program in Bioengineering Seoul National University Seoul Republic of Korea; ^2^ SKKU Advanced Institute of Nanotechnology (SAINT) Sungkyunkwan University Suwon South Korea; ^3^ Nanophotonic System Research Center Korea Institute of Science and Technology (KIST) Seoul Republic of Korea; ^4^ KU‐KIST Graduate School of Converging Science and Technology Korea University Seoul Republic of Korea; ^5^ School of Chemical and Biological Engineering Institute of Chemical Processes Seoul Republic of Korea; ^6^ School of Biomedical Engineering Korea University Seoul Republic of Korea; ^7^ Department of Micro/Nano Systems Korea University Seoul Republic of Korea; ^8^ Department of Nano Science and Technology Sungkyunkwan University Suwon South Korea; ^9^ Department of Nano Engineering Sungkyunkwan University Suwon South Korea

**Keywords:** date rape drug, drug‐facilitated sexual assault, drug‐related crime, self‐protection, γ‐hydroxybutyric acid

## Abstract

Drug misuse and abuse remain critical global challenges, driving public health and societal burdens through increased morbidity and mortality, impaired driving, interpersonal violence, and escalating healthcare and criminal‐justice expenditures. Drug‐facilitated sexual assault (DFSA) remains a pressing societal concern, and γ‐hydroxybutyrate (GHB) is a representative DFSA agent categorized as a date rape drug. GHB is endogenously derived, exists at low concentrations, and has a short detection window and weak optical features that complicate conventional assays and contribute to missed or delayed detection. These challenges are especially important because GHB can induce rapid clinical presentation that overlaps with alcohol or other sedatives, making timely identification critical for acute management and safeguarding decisions. This necessitates reliable detection of exogenous GHB in minimally invasive samples. This study presents a graphene‐based electrical biosensor for rapid detection of GHB with high sensitivity and selectivity, achieving a limit of detection (LOD) of 100 fm for standard GHB solutions and a correlated LOD of 100 fm in artificial urine matrices. The platform enables rapid, on‐site detection with concentration‐dependent electrical responses across a wide dynamic range suitable for clinically and forensically relevant GHB levels, with broad implications for diagnostics, forensic analysis, and public health strategies to reduce drug‐related harm.

## Graphical abstract

A graphene‐based electrical biosensor enables rapid and highly sensitive detection of GHB at 100 fm in both standard solutions and artificial urine, providing reliable point‐of‐need screening for drug‐facilitated sexual assault. The platform demonstrates strong selectivity, fast response, and a wide dynamic range, supporting accurate detection in complex biological environments for clinical and forensic applications.

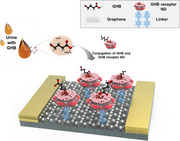

## 1 Introduction

Drug misuse and abuse are critical global challenges driving substantial public health and societal burdens through increased morbidity and mortality, impaired driving, interpersonal violence, and escalating healthcare and criminal‐justice expenditures [1, 2, 3]. The prevalence of drug‐facilitated sexual assaults (DFSA) is currently high, with women as the main target and leading to date rape [4, 5]. γ‐hydroxybutyrate (GHB) is a representative DFSA categorized as a date rape drug (DRD) [6, 7, 8, 9]. GHB is endogenously derived (existing at low concentrations) and has a short detection window and weak optical features that complicate conventional assays and contribute to missed or delayed detection. In forensic contexts, endogenous GHB concentrations in blood are typically very low, generally ranging from the limit of detection up to approximately 2–6 nmol mL^−1^, whereas exogenous GHB intake results in markedly elevated plasma levels, often exceeding 200–400 nmol mL^−1^ depending on dose and timing after ingestion [10]. Therefore, a platform that can rapidly detect GHB directly from minimally processed saliva or urine could address high‐impact, real‐world cases while providing a scalable foundation for broader drug panels as GHB intake has a high possibility of inducing rapid clinical presentation, which might overlap with that of alcohol or other sedatives. Additionally, timely identification can meaningfully influence acute management, monitoring, and safeguarding decisions. This warrants reliable detection of exogenous GHB in minimally invasive samples in settings where rapid documentation of exposure is needed given that DRD use is one of the crucial social issues.

Rapid and reliable identification of intoxicants at the point of need is central to effective acute medical care, harm‐reduction practice, and time‐sensitive law‐enforcement decision‐making [8, 11, 12, 13]. However, the analytical methods widely used in clinical and forensic toxicology still face challenges. Immunoassays and colorimetric screens are fast and inexpensive, but frequently suffer from limited analyte coverage and false positives/negatives due to cross‐reactivity and matrix interference [14, 15]. To address these limitations, recent studies have explored electrochemical sensing approaches for the rapid screening of date rape drugs, however, GHB remains one of the least investigated targets due to its poor electrochemical behavior and matrix‐related challenges [16]. Conversely, confirmatory liquid chromatography–mass spectrometry (LC–MS) delivers high specificity and broad scope, but typically requires specialized infrastructure, trained personnel, and lengthy sample‐to‐answer workflows that constraint utility in frontline settings, such as emergency departments, roadside and custodial checkpoints, shelters, harm‐reduction clinics, and disaster response operations [17]. These environments impose stringent requirements on sensing platforms, with results required to be generated within a short time period from small‐volume samples with minimal preparation, while maintaining sensitivity and selectivity in complex biological matrices. Consequently, there is an urgent need for analytical systems that are not only high‐performing and field‐deployable, but also readily updatable with the capability of incorporating new molecular targets without complete redesign of the device or workflow. In this context, graphene field‐effect transistor (GFET)–based sensing offers distinct advantages over existing colorimetric GHB screening kits, including label‐free electrical readout, reduced susceptibility to optical interference from complex matrices, and enhanced sensitivity at low analyte concentrations. A systematic comparison with representative GHB detection methods is summarized in Table S1, providing an overview of the key advantages and limitations of the proposed platform.

In this study, a graphene‐based electrical biosensor for the rapid detection of GHB with high sensitivity and selectivity is presented. It is recognized that GFET‐based biosensing in biological fluids is inherently challenged by Debye screening effects, particularly in high ionic strength media such as saliva, where electrostatic interactions are strongly screened. The system integrates receptor‐embedded nanodiscs (NDs) immobilized on a graphene field‐effect transistor platform, which enables a molecular recognition event to be transduced into a stable, quantitative electrical signal within a few seconds.

Here, we use a GHB receptor (GHBR) protein, a predicted multi‐pass membrane protein comprising approximately 10–11 transmembrane helices, reconstituted in NDs to preserve its native‐like membrane environment. This receptor‐based strategy was chosen over antibodies or aptamers because GHB is a small endogenous metabolite that is difficult to target with high affinity and selectivity, whereas a native receptor architecture enables intrinsically specific molecular recognition and stable electrical transduction in complex biological matrices [18]. We propose that immobilization of GHBR‐embedded NDs on a GFET enables rapid and selective electrical transduction of GHB binding, allowing sensitive detection at clinically relevant concentrations even in high‐ionic‐strength biological fluids. To achieve robust performance in operationally relevant matrices, the platform combines interference‐resilient readout with onboard signal deconvolution designed to mitigate matrix effects and reduce susceptibility to cross‐reactivity. Beyond analytical performance, the platform is designed with translational constraints that ultimately govern real‐world impact. By enabling rapid, on‐site detection within clinical and justice‐system workflows, the approach shortens time‐to‐result and can reduce reliance on delayed, resource‐intensive when immediate decisions are required [19]. Moreover, rapid identification of GHB exposure aligns with the evolving complexity of contemporary substance‐use patterns where co‐ingestion with alcohol and other depressants is common and supports earlier recognition for triage, risk stratification, and downstream intervention. Collectively, these results position graphene‐based biosensing as a modular and adaptable foundation for next‐generation chemical surveillance and decision‐support technologies, with broad implications for diagnostics, forensic analysis, and public health strategies aimed at reducing drug‐related harm.

## 2 Results and discussion

### 2.1 Overall Concept of the Study

**FIGURE 1:**
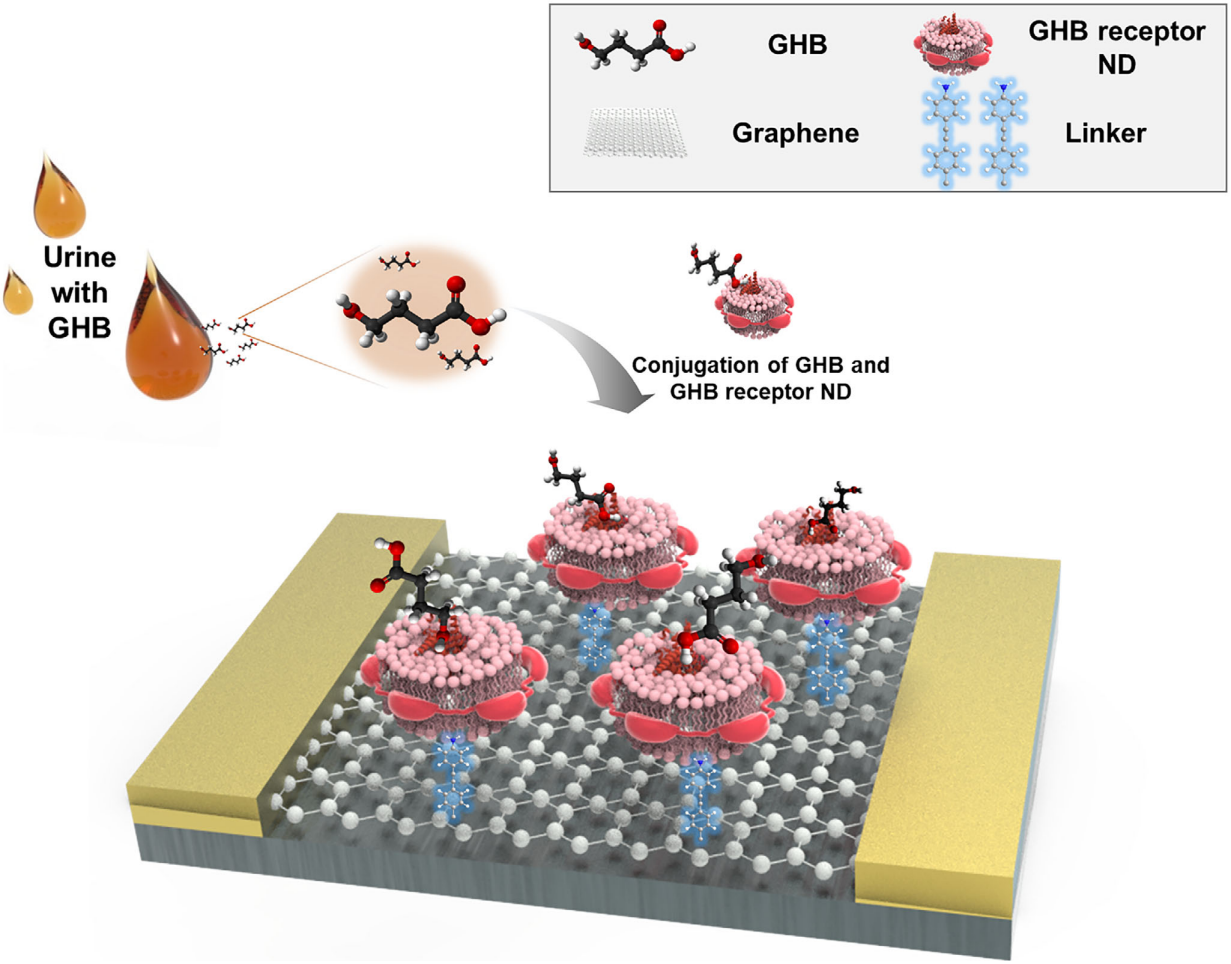
Overall concept of the study.

Figure 1 presents a schematic overview of the graphene‐based biosensing platform in which GHB receptor NDs (GHBR NDs) are immobilized onto a graphene transducer to create a stable, bioactive recognition interface. Upon exposure to the sample, GHB interacts specifically with the receptor sites embedded in the NDs, forming receptor–analyte complexes at the graphene surface. These binding events modulate the local charge environment and interfacial electrical properties of graphene, producing a measurable change in the electrical signal that correlates with the presence and concentration of the target. Overall, the schematic highlights how coupling receptor‐embedded NDs with a highly sensitive graphene transducer enables selective detection through direct transduction of molecular recognition into an electrical signal.

Importantly, the platform demonstrates the capability to detect GHB in urine matrices. Feasibility was validated using artificial urine to confirm the performance and reliable signal transduction in physiologically relevant conditions. By integrating receptor‐embedded NDs, this platform enables highly selective detection through direct electrical transduction of molecular binding events, highlighting its potential for practical bioanalytical and clinical applications.

### 2.2 Fabrication of NDs

**FIGURE 2:**
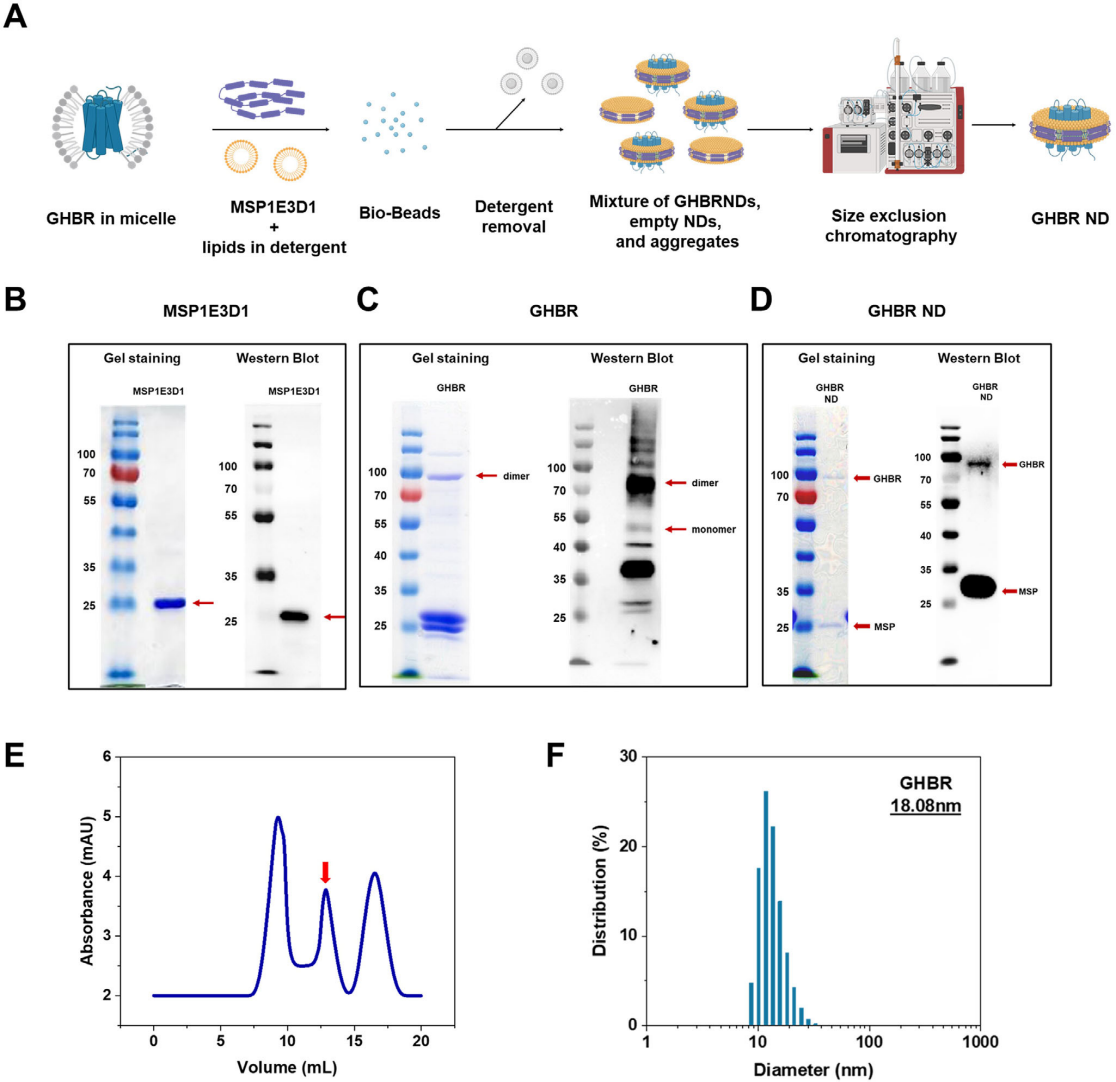
Characterization of GHBR‐embedded ND. (A) Schematic illustration of the general workflow showing the production process of GHBR‐embedded NDs. The GHBR were expressed from *E.coli*. (B) SDS‐PAGE and Western blot analyses of MSP1E3D1 produced in *E.coli*. (C) SDS‐PAGE and Western blot analyses of purified GHBR expressed in *E.coli*. The band of (D) SDS‐PAGE and Western blot analyses of the purified GHBR‐NDs. (E) Size‐exclusion chromatogram of GHBR ND. (F) Dynamic light scattering (DLS) results of GHBR ND.

GHBR was produced in *Escherichia coli* and reconstituted into ND structures to provide a stable membrane‐mimicking environment. The overall procedure for GHBR ND production is illustrated in Figure 2a. Specifically, 1,2‐Dimyristoyl‐sn‐glycero‐3‐phosphocholine (DMPC) was first mixed with the membrane scaffold protein MSP1E3D1, and purified GHBR in detergent micelles was then added at an optimized molar ratio of GHBR:MSP1E3D1: DMPC = 1:2:300, followed by detergent removal using Bio‐Beads to induce self‐assembly of GHBR‐embedded NDs. Since membrane proteins, such as GHBR, are highly hydrophobic and often unstable in aqueous environments when maintained only in detergent micelles, the ND platform was adopted to provide a lipid bilayer tightly wrapped by MSP1E3D1, thereby mimicking the native membrane structure and improving receptor stability in solution [20, 21, 22]. For ND assembly, MSP1E3D1, a membrane scaffold protein derived from human apolipoprotein A‐I, was expressed in *E. coli* and purified, showing a band at approximately 26 kDa in both sodium dodecyl sulfate (SDS)‐polyacrylamide gel electrophoresis (PAGE) gel staining and western blot analysis (Figure 2b), which confirmed successful preparation of the scaffold protein capable of effectively wrapping lipid/receptor complexes [23]. GHBR was expressed in *E. coli* as inclusion bodies, solubilized using SDS and dithiothreitol (DTT), and purified by affinity chromatography on a Ni^2^^+^‐NTA column, and its expression and purification were confirmed by SDS‐PAGE and western blotting (Figure 2c). To further verify GHBR expression and purification at each step, detailed analyses corresponding to individual expression and purification steps were performed (Figure S1). Original uncropped Western blot images corresponding to the data presented in Figure 2B–D and Figure S1 are provided in Figures S2–S5. Although the theoretical molecular weight of GHBR is 45.7 kDa, GHBR was predominantly detected in a dimeric form in both analyses, showing an apparent molecular weight approximately twice that of the monomer. After ND assembly, the formation of GHBR‐embedded NDs was verified by SDS‐PAGE gel staining and western blot analysis of the purified ND fraction, where both GHBR and MSP1E3D1 bands were clearly observed, confirming successful incorporation of GHBR into ND structures (Figure 2d). The synthesized GHBR NDs were separated from aggregates by size exclusion chromatography (SEC). Figure 2e shows the SEC elution profile of the produced GHBR NDs, where the GHBR ND fraction was clearly separated from the aggregation peak. Figure 2f shows dynamic light scattering (DLS) analysis results of the purified GHBR NDs, exhibiting a narrow and uniform size distribution with an average hydrodynamic diameter of approximately 18.08 nm, indicating homogeneous formation of GHBR NDs with high quality.

### 2.3 Surface Analysis

**FIGURE 3:**
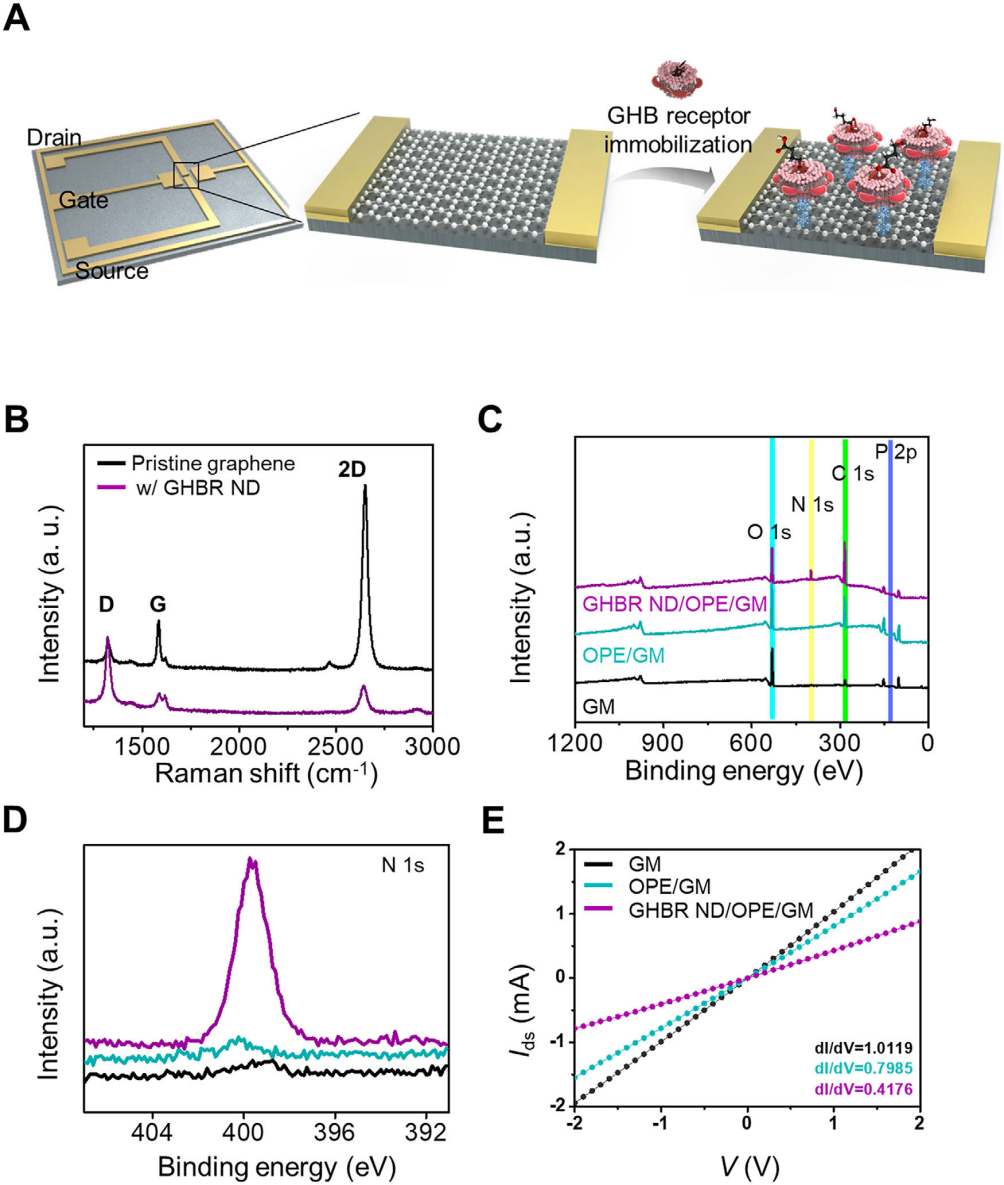
Characterization of GHBR NDs immobilized on graphene‐based biosensor. (A) Schematic illustration of GHBR NDs functionalization. (B) Raman spectra of bare graphene, immobilized with OPE, and GHBR NDs in consecutive order. (C) Survey data and (D) narrow XPS of bare graphene, immobilized with OPE, and GHBR NDs. (E) *I‐V* curves for each immobilization steps of the surface modification.

A schematic illustration of the side‐gated FET detection system is shown in Figure 3A. The electrode shows a significant change in current when the target biomarker GHB is injected into the chamber. To confirm proper immobilization of NDs on the graphene channel, Raman spectroscopy was performed (Figure 3B). The G peak and 2D peak were observed at 1587 and 2651 cm^−1^, respectively. A pristine graphene (black line), appearing as a monolayer where the ratio of *I*_2D_/*I*_G_ was approximately 1.97 [24, 25, 26]. The D band at 1326 cm^−^^1^ from vibrations of sp^2^ carbon rings, was observed, suggesting the presence of defects consistent with disrupted covalent bonding between oligo(phenylene‐ethynylene) (OPE)/GHBR NDs and graphene in the OPE/GHBR ND‐conjugated graphene. In this system, tetrabutylammonium fluoride (TBAF) was employed as an efficient desilylation reagent to activate linker molecules prior to their immobilization onto the graphene channel. The linker molecules were initially protected with silyl groups to enhance their chemical stability and to suppress undesired side reactions during multistep synthesis and handling. These protecting groups effectively masked reactive functionalities, such as hydroxyl, amine, or carboxyl groups, thereby maintaining the chemical inertness of the linkers until the desired stage of surface functionalization. Upon treatment with TBAF, fluoride ions (F^−^), owing to their strong affinity for silicon and the high thermodynamic stability of the Si─F bond, selectively cleaved Si─C or Si─O bonds via nucleophilic attack. This process proceeded under mild, anhydrous conditions, enabling efficient removal of the silyl protecting groups without perturbing other sensitive functionalities within the linker molecules. As a result, the previously masked reactive moieties were exposed in their active forms. This activation step is critical for generating chemically accessible termini capable of interacting with the graphene surface. Such interactions may occur through covalent bonding at intrinsic defect sites, edge planes, or pre‐functionalized regions of the graphene channel. Notably, the use of TBAF allows this transformation to proceed under conditions that preserve the structural and electronic integrity of graphene. Consequently, this strategy enables precise and controlled immobilization of linker molecules, thereby facilitating subsequent functionalization while maintaining the intrinsic properties of the graphene‐based system [27]. In addition, characteristic amide I and amide II bands were detected at 1623–1634 cm^−^^1^ and 1234–1286 cm^−^^1^, respectively [27, 28, 29]. Moreover, x‐ray photoelectron spectroscopy (XPS) scan was performed to detect the major core level of O 1s (532.5 eV), N 1s (400.3 eV), C 1s (284.4 eV), and P 2p (128.8 eV). No impurity‐related core‐level signals were detected in the survey spectra (Figure 3C) [30, 31, 32]. After functionalization of the micropatterned graphene (GM) with OPE bearing a terminal amine group, a distinct N 1s signal emerged at 400.3 eV, confirming the successful incorporation of OPE/GHBR NDs (Figure 3D). To verify successful surface functionalization, the electrical characteristics of OPE‐ and OPE/GHBR ND‐conjugated graphene were evaluated by generating a current–voltage (*I–V*) curve under conditions of voltage of −2–2 V and scan rate of 0.1 V ms^−^^1^ (Figure 3E). The conductivity (d*I*/d*V*) was calculated as the slope: 1.0119 for GM, 0.7985 for OPE/GM, and 0.4176 for GHBR NDs/OPE/GM. Although surface modification of GM led to a measurable decrease in its electrical conductivity due to the introduction of additional interfacial scattering sites and disruption of the native charge‐transport pathways, a stable Ohmic contact was still maintained after ND functionalization [33, 34, 35, 36]. Notably, the *I–V* curve characteristics remained linear over the investigated bias range, indicating that the contact resistance did not become dominated by a Schottky‐type barrier and that efficient carrier injection/extraction was preserved despite the added surface chemistry. These results suggest that ND functionalization modifies the transport properties of GM without compromising the fundamental Ohmic nature of the electrical interface, supporting the robustness of the contact for subsequent device operation and characterization [37, 38].

### 2.4 GHB Detection

**FIGURE 4:**
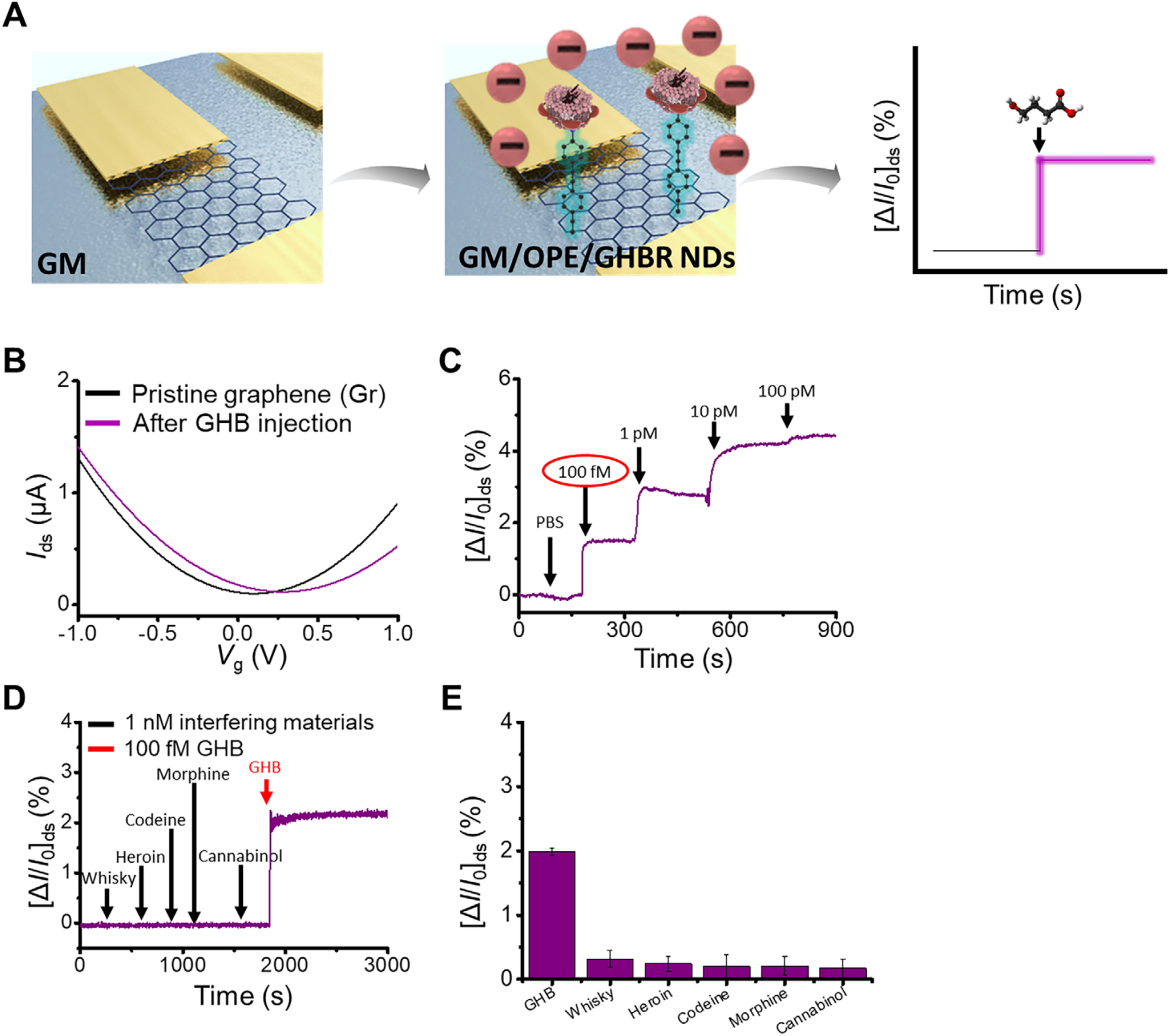
Electrical Performance of GHB Detection (A) Schematic illustration of GHB detection graphene‐based biosensor mechanism. (B) Transfer curve of before and after GHB injection. (C) Real‐time monitoring graph of GHB from 100 fm to 100 pm. (D) Selectivity test of GHB detection graphene‐based biosensor with other higher concentration interfering materials and the target. (E) The normalized sensor responses to GHB and various potential interfering analytes are compared.

Figure 4 presents real‐time electrical responses obtained upon exposure to GHB standard samples, demonstrating the capability of the platform to continuously track analyte‐induced signal changes with high sensitivity. Figure 4A schematically illustrates the sensing mechanism of the GHB‐functionalized GFET. The graphene channel was functionalized with GHB‐specific recognition elements, enabling selective binding of GHB molecules at the graphene surface. Upon exposure to GHB, specific interactions between GHB and the surface‐immobilized receptors induce local charge redistribution in close proximity to the graphene channel. This interfacial charge modulation alters the carrier density in graphene, leading to measurable changes in the electrical characteristics of the GFET, such as shifts in the Dirac point and variations in the drain current. Because graphene is atomically thin and highly sensitive to surface charge perturbations, even subtle molecular binding events occurring within the Debye screening length can be efficiently transduced into electrical signals. As a result, the GHB detection graphene‐based biosensor enables real‐time, label‐free detection of GHB with high sensitivity. The magnitude of the electrical response directly correlates with the amount of GHB bound to the sensor surface, forming the basis for quantitative real‐time monitoring across a wide concentration range. Figure 4B shows the representative transfer characteristics of the GHB detection graphene‐based biosensor before and after exposure to GHB. Upon introduction of GHB, a distinct shift in the Dirac point was observed, indicating effective modulation of the graphene channel conductivity induced by GHB binding. The magnitude and direction of the Dirac point shift reflect changes in carrier density arising from GHB detection graphene‐based biosensor interactions at the graphene surface, confirming that the GFET transduces molecular recognition events into measurable electrical signals. The sensing mechanism of the graphene‐based platform is primarily by the adsorption‐induced charge transfer between GHB molecules and the graphene surface. The high carrier mobility and atomically thin structure of graphene show high sensitivity to electronic uncertainty arising from molecular adsorption. GHB molecules contain hydroxyl and carboxyl functional groups that facilitate their interaction with the π‐conjugated graphene lattice via weak van der Waals interactions and possible hydrogen bonding. Upon adsorption, these functional groups promote charge transfer at the graphene interface which leads to a redistribution of charge carriers within the graphene channel. This process distracts the Fermi level and consequently modulates the electrical conductivity of the device. The charge‐transfer process is further supported by the transfer characteristics which exhibit a distinct shift in the Dirac point upon exposure to GHB. This shift indicates doping of the graphene channel induced by molecular adsorption. The observed direction of the shift suggests p‐type doping behavior, which can be attributed to the interaction between GHB molecules and the graphene π‐electron system that results to an effective increase in hole carrier concentration.

The real‐time electrical responses of the GHB–GFET sensor to stepwise increases in GHB concentration are presented in Figure 4C. Sequential injections of GHB resulted in immediate and well‐defined changes in the normalized drain current (Δ*I*/*I*_0_), demonstrating rapid sensor response and stable signal evolution over time. Notably, the signal intensity increased with increasing GHB concentrations, confirming a concentration‐dependent response behavior. Even at ultralow concentrations, clear signal changes were observed relative to the baseline, highlighting the high sensitivity of the GFET platform for real‐time GHB monitoring. Higher concentration of GHB solution was also tested (Figure S6) and it was shown that the GHB‐GFET sensor platform is able to detect as high as 100 mm in few second.

The selectivity of the presented GHB‐GFET sensor was evaluated by comparing its response to GHB against those elicited by potential interfering species. These included representative compounds as well as an alcohol‐based substance commercial whisky to simulate DFSA‐relevant interference. The results demonstrate that the sensor exhibits a markedly higher response to GHB which confirms the high selectivity of the GHB‐GFET sensor (Figure 4D). While GHB induced a pronounced change in the normalized current (Δ*I*/*I*_0_), negligible responses were observed for other tested analytes, even at higher concentrations. This distinct response difference demonstrates the high selectivity of the sensor toward GHB and confirms that the observed electrical signals originate from specific GHBR interactions rather than nonspecific adsorption or background effects. Furthermore, the normalized response to GHB is remarkably higher compared to the non‐interfering materials (Figure 4E). This indicates that the sensor maintains high fidelity even in complex chemical environments containing ethanol and other organic constituents. The low response variance among interfering species suggests excellent non‐interference capability which is critical for real‐world applications such as forensic analysis and on‐site screening. The ability to discriminate GHB from alcohol‐containing beverages is particularly important, as ethanol‐rich environments represent a major challenge in DFSA detection scenarios. Overall, these findings confirm that the GHB–GFET sensor demonstrates exceptional selectivity, positioning it as a promising platform for reliable and sensitive detection of GHB in complex matrices.

### 2.5 GHB Detection in Artificial Urine

**FIGURE 5:**
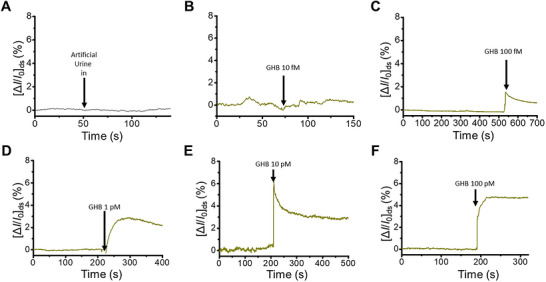
Real‐time monitoring results of GHB in artificial urine and various concentration from (A) Artificial urine sample, (B) 10 fm GHB in artificial urine, (C) 100 fm GHB in artificial urine, (D) 1 pm GHB in artificial urine, (E) 10 pm GHB in artificial urine, and (F) 100 pm GHB in artificial urine.

Figure 5 shows the real‐time monitoring results for GHB detection in artificial urine. Artificial urine was employed as a representative physiological matrix to evaluate sensor performance under controlled yet physiologically relevant conditions, enabling reproducible assessment while minimizing interference from endogenous GHB. Different concentrations of GHB in artificial urine were prepared, ranging from 10 fm to 100 pm, and were monitored individually. Each concentration was prepared independently and analyzed under identical experimental conditions to ensure reproducibility and minimize systematic variation. As shown in Figure 5A, artificial urine did not introduce measurable background noise or nonspecific interference to the sensing platform. The baseline remained stable throughout the monitoring period, and no significant noise was detected. The resulting time‐resolved response profiles demonstrate clear, concentration‐dependent signal evolution across the entire dynamic range, confirming the capability and reliability of the platform to detect low concentrations of GHB in a biomimetic environment. When 10 fm GHB in artificial urine was monitored (Figure 5B), no significant electrical signal was observed. However, upon injection of 100 fm GHB in artificial urine into the platform, a clear electrical signal was detected, as shown in Figure 5C. Therefore, the LOD of GHB in artificial urine was determined to be 100 fm, which is comparable to the LOD obtained using standard GHB solutions in the range of 1–100 pm (Figure 5D–F). This result is particularly significant because matrix components in biological mimetic fluids often compromise sensitivity due to nonspecific adsorption, competitive binding, or signal damping effects. The preservation of the 100 fm LOD in artificial urine indicates that the sensing interface maintains its intrinsic analytical sensitivity even in the presence of salts, urea, creatinine, and other constituents that mimic real urinary composition. In addition to maintaining the LOD, quantitative comparison between artificial urine samples and standard solutions revealed a strong correlation in signal intensity, as shown in Figure 4. These results demonstrate that the proposed sensing platform achieves ultrahigh sensitivity (LOD = 100 fm), excellent matrix tolerance, insignificant background interference, and strong quantitative correlation between standard and artificial urine samples, with a detection range that is well below endogenous urinary GHB levels (0–1.5 mg/L) and far lower than concentrations observed after exogenous intake, which can reach several hundred to several thousand mg/L [39, 40, 41]. In practical applications, sensor readouts can be interpreted relative to established concentration thresholds, enabling differentiation between endogenous background levels and exogenous GHB exposure.

## 3 Conclusions

This study presents a highly validated and extensible graphene‐based sensing platform that addresses key limitations inherent in current clinical and forensic drug detection methodologies. By integrating receptor‐embedded ND assemblies with GFET architectures, the system enables rapid, sensitive, and selective detection of GHB directly from minimally processed biological matrices, including artificial urine, with a LOD of 100 fm and a response time of a few seconds. Operational assessments highlighted the platform's suitability for application in distributed, point‐of‐need environments where time‐critical decision‐making is essential. Importantly, the modular receptor interface supports straightforward assay tailoring and optimization for GHB measurement across different sample types and use scenarios, while interference‐resilient signal processing and ambient stability help maintain performance in complex biological backgrounds. Together, these attributes underscore the platform's potential as a scalable solution for real‐time GHB monitoring across diverse clinical, forensic, and public health contexts. Notably, the achieved sensitivity enables detection of GHB at concentrations well below reported endogenous background levels, supporting identification of exogenous exposure within the clinically relevant window following ingestion, including the early hours when safeguarding, triage, and risk stratification decisions are most critical. In practical use, quantitative readings from the GHBR‑ND–GFET would be interpreted relative to established urinary cut‑off concentrations and the time of sampling, such that values below the cut‑off are considered compatible with endogenous background, whereas values exceeding the cut‑off within the typical detection period support exogenous GHB exposure. Overall, this work establishes a unified framework that bridges advanced materials engineering with translational sensing applications, positioning graphene‐based GFET sensing as a next‐generation foundation for chemical surveillance, risk stratification, and evidence‐based intervention for GHB‐related intoxication and exposure. Future research will focus on further improving the GHB assay performance, enhancing miniaturization, and integrating low‐power wireless communication modules, such as Bluetooth Low Energy (BLE) or near‐field communication (NFC), with defined power‐consumption targets, to enable portable and semi‐autonomous field deployment in both resource‐rich and resource‐limited settings.

## 4 Experimental section

### 4.1 Fabrication of Drug Detection Electrodes

The side‐gated graphene chip (8 cm × 8 cm) was fabricated using the chemical vapor deposition method and transferred on to a silicon dioxide (SiO_2_) wafer through the wet transfer method. The microelectromechanical systems (MEMS) process was used to fabricate the electrodes for receptonics. The MEMS process includes the spin‐coating of photoresist (GXR‐601, AZ electronic materials, and DNR L300‐40, Dongjin Semichem Co., Ltd.), reactive‐ion etching (OXFORD ETCHER 80PLUS, Oxford Instrument) for graphene micropatterning, and photolithography (MA‐6, Karl‐Suss MicroTec) for electrode patterning. Cr/Au (10/100 nm) was deposited using an electronic‐beam (E‐beam) evaporator (ZZS550‐2/D, MAESTECH) to form electrodes. Then, the electrodes were passivated with 50 nm of SiO_2_ to prevent non‐specific binding, followed by electrode dicing (DAD 3350, Disco).

### 4.2 Immobilization of Linkers and Bioprobe on the Electrode Graphene Channel

To perform the surface treatment of electrodes, 4 µL of 2 µm OPE and 2 µm tetrabutylammonium fluoride solution were dropped onto the graphene channel portion of the electrode in this order. After 30 min, each electrode was rinsed with distilled water. The electrode was conjugated with glutaraldehyde (coupling agent) for 4 h. Then, GHBR NDs were immobilized on the graphene channel by incubation at 4°C for 4 h.

### 4.3 Characterization of NDs

FT‐IR was conducted using Alpha‐P instrument after each surface treatment procedure. Raman spectra were obtained using a Raman spectrometer (LabRAM HR Evolution Visible_NIR, HORIBA, Japan); a 633 nm visible laser was focused on the samples. XPS spectra were measured using a Sigma Probe (Axis‐Supra, Kratos) to provide further proof of immobilization.

### 4.4 Sensing Performance

The sensing performance of the receptonics platform was measured using standard samples and Keithley 2612A source meter, and the change in current was normalized using the following Equation (1):

(1) $$\Delta I/I_{0}=\left(I-I_{0}\right)/-I_{0}$$

where *I*_0_ is the initial current, and *I* is the current measured at a given timepoint during real‐time monitoring.

### 4.5 Expression and Purification of GHBR

A bacterial expression vector encoding GHBR was synthesized and inserted into the pET‐IDT‐C His vector (Integrated DNA Technologies, USA). To enhance GHBR production, the *RraA* gene in the pBAD33.1 plasmid was co‐expressed, which is known to increase protein yield by inhibiting *E. coli* RNase E‐mediated mRNA degradation.

*E.coli* strain BL21 (DE3) (Real Biotech Corporation, Taiwan) was co‐transformed with two plasmids containing GHBR and RraA genes, respectively. Transformants were plated on pre‐warmed Luria‐Bertani (LB) agar containing kanamycin (50 µg/mL) and chloramphenicol (40 µg/mL), followed by overnight incubation at 37°C. A single colony was inoculated into 5 mL LB and cultured at 37°C for 6 h in an orbital shaker, then transferred into 250 mL LB and grown overnight. The culture was transferred to 6 L of LB medium and incubated at 30°C and 150 rpm until the OD_600_ value reached 0.4–0.5. The culture was induced with 0.1 mm isopropyl β‐D‐thiogalactoside and 0.2% L‐arabinose, and incubation was continued overnight at 25°C to ensure complete induction. Cells were harvested by centrifugation at 7000 rpm for 10 min at 4°C and resuspended in PBS containing 2 mm ethylenediaminetetraacetic acid (EDTA). After adding a protease inhibitor cocktail (GenDEPOT, USA), the cells were disrupted on ice by sonication for 5 min at 38% amplitude using 5 s on and 5 s off cycles. The lysate was centrifuged at 13 000 rpm for 30 min at 4°C. The insoluble cellular lysate was solubilized in solubilization buffer (0.1 m Tris‐HCl, 20 mm SDS, 100 mm DTT, 1 mm EDTA, pH 7.4) and incubated at 30°C with shaking at 120 rpm overnight. Subsequently, solubilized proteins were centrifuged at 12 000 rpm for 30 min at 25°C and then the supernatant sample was dialyzed using a 10 kDa MWCO dialysis cassette (Thermo Scientific) against dialysis buffer (100 mm sodium phosphate, 10 mm SDS, pH 8.0) overnight. Prior to affinity purification, the sample was filtered using a 0.22 µm bottle‐top filter (Sartorius, Germany) and loaded onto a HisTrap HP column (Cytiva, USA) equilibrated with binding buffer (100 mm sodium phosphate, 10 mm SDS, pH 8.0). After sample loading, the column was washed using a pH gradient from pH 8.0 to 7.0, and GHBR was eluted with buffer at pH 6.0. The eluted fraction was subsequently exchanged into hydroxyethyl piperazine Ethane Sulfonic acid (HEPES) buffer I (20 mm HEPES‐NaOH, 100 mm NaCl, 20 mm cholate, pH 8.0) using a HiTrap Desalting column (Cytiva, USA). All purification steps were performed using an AKTA pure FPLC system.

### 4.6 Expression and Purification of MSP1E3D1

MSP1E3D1 was expressed using the pMSP1E3D1 bacterial expression vector. Following protein expression, cells were harvested by centrifugation at 7000 rpm for 10 min at 4°C and resuspended in lysis buffer (20 mm Tris‐HCl, 20 mm imidazole, and 0.5 m NaCl, pH 8.0). The resuspended cells were disrupted by sonication for 5 min using 5 s on/off cycles. After lysis, cell debris was removed by centrifugation at 12 000 rpm for 30 min at 4°C. The supernatant containing soluble MSP1E3D1 was then filtered using a 0.45 µm bottle‐top filter and loaded onto a HisTrap HP affinity column using an FPLC system. The column was sequentially washed with washing buffer (20 mm Tris‐HCl, 50 mm imidazole, and 0.5 m NaCl, pH 8.0), and MSP1E3D1 was eluted using elution buffer (20 mm Tris‐HCl, 350 mm imidazole, and 0.5 m NaCl, pH 8.0). Finally, the purified MSP1E3D1 was buffer exchanged into HEPES buffer I using a HiTrap HP desalting column and stored at −80°C until further use.

### 4.7 Reconstitution of Purified GHBRs Into NDs

DMPC (Avanti Polar Lipids, AL, USA) was used as the lipid for ND formation. Lipids were dried using nitrogen gas from a chloroform solution and vacuumed overnight to remove residual chloroform. The dried lipid was then solubilized in cholate buffer (20 mm HEPES, 100 mm NaCl, 1 mm EDTA, 100 mm cholate, pH 8.0). For GHBR ND reconstitution, purified GHBR, MSP, and DMPC were mixed at a molar ratio of 1:2:300 and incubated at 25°C for 2 h with gentle agitation. Bio‐Beads (Bio‐Rad, USA) were added to this mixture to remove the detergents at 25°C with overnight agitation. After removing the Bio‐Beads, the sample was further purified using a Superdex 200 Increase 10/300 GL SEC column (Cytiva, USA) with HEPES buffer II at a flow rate of 0.75 mL/min to separate GHBR NDs from larger aggregates and smaller protein impurities. Collected fractions were evaluated by SDS‐PAGE and western blotting.

### 4.8 SDS‐PAGE and Western Blot Analysis

GHBR bacterial lysates and GHBR NDs were mixed with 4× Laemmli sample buffer (Biorad, USA) and heated at 95°C for 5 min. The prepared samples were loaded onto a 10% polyacrylamide gel and electrophoresed at 100 V in SDS running buffer. The gels were stained overnight at room temperature with Coomassie Brilliant Blue R‐250 staining solution (Biosesang, South Korea). Destaining was performed using destaining solution (Biosesang, South Korea) until distinct protein bands were observed.

For western blotting, proteins were transferred onto a PVDF membrane (Cytiva, USA) at 4°C. The membrane was blocked with EveryBlot blocking buffer (Bio‐Rad, USA) for 20 min at room temperature. The primary and secondary antibodies used were anti‐His probe mouse antibody and HRP‐conjugated anti‐mouse antibody, respectively. Chemiluminescent signals were developed using an enhanced chemiluminescence (Ab Frontier, Korea) detection kit and imaged using a ChemiDoc imaging system (Bio‐Rad, USA).

### 4.9 DLS Analysis

Size distribution of purified GHBR NDs was characterized by DLS using a Zetasizer Nano ZSP instrument (Malvern, UK). For analysis, 1 mL of ND sample was transferred into a disposable cuvette. After an equilibration period of 120 s, measurements were performed three times.

## Author Contributions

**Jai Eun An**: conceptualization: (lead); data curation: (lead); formal analysis: (lead). **Jisung Kwak**: data curation: (equal); formal analysis: (equal). **Kyung Ho Kim**: formal analysis: (supporting). **Chaeeun Kim**: formal analysis: (supporting). **Sung Eun Seo**: investigaion: (supporting). **Nathaniel S. Hwang**: investigation: (supporting). **Yong‐Sang Ryu**: investigation: (supporting). **Hyun Seok Song**: conceptualization: (equal); investigation: (equal); writing – review & editing: (equal). **Oh Seok Kwon**: conceptualization: (lead); funding acquisition: (lead); writing – review & editing: (lead).

## Conflicts of Interest

The authors declare no conflicts of interest

## Supporting information




**Supporting File**: advs76399‐sup‐0001‐SuppMat.docx.

## Data Availability

The data that supports the findings of this study are available in the supplementary material of this article.
