# Supplementary material for: An Immediate‐Response Detection System for γ‐Hydroxybutyrate to Enhance Personal Safety in Social Environments
Source: Adv Sci (Weinh). 2026 Jul 6:e76399. Online ahead of print. doi: 10.1002/advs.76399 (PMC13336012; doi:10.1002/advs.76399)
Supplement: Supplementary file 1 — Supporting File: advs76399‐sup‐0001‐SuppMat.docx. [file ADVS-9999-e76399-s001.docx]

Supporting Information

**An Immediate-Response Detection System for γ-Hydroxybutyrate to Enhance Personal Safety in Social Environments**

Jai Eun An,^a,b,†^ Jisung Kwak,^c,†^, Kyung Ho Kim ^b^, Chaeeun Kim _,_^c^ Sung Eun Seo ^b^, Nathaniel S. Hwang^a,d^, Yong-Sang Ryu^e^_,_ Hyun Seok Song,^c,*^ Oh Seok Kwon ^b,,f,g^*

**Table S1**. Systematic comparison of representative GHB detection methods and the GHBR‑ND‑GFET platform


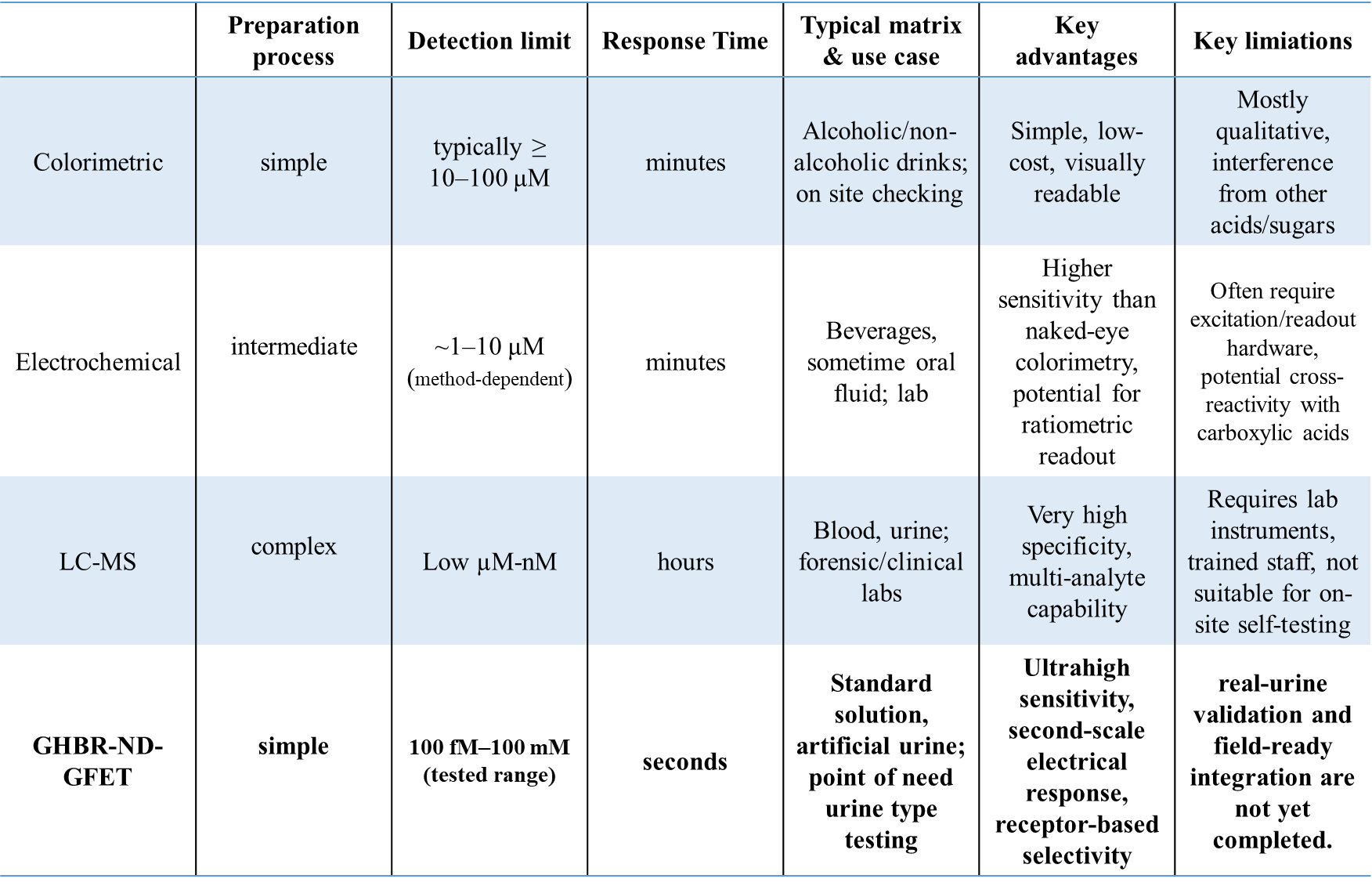


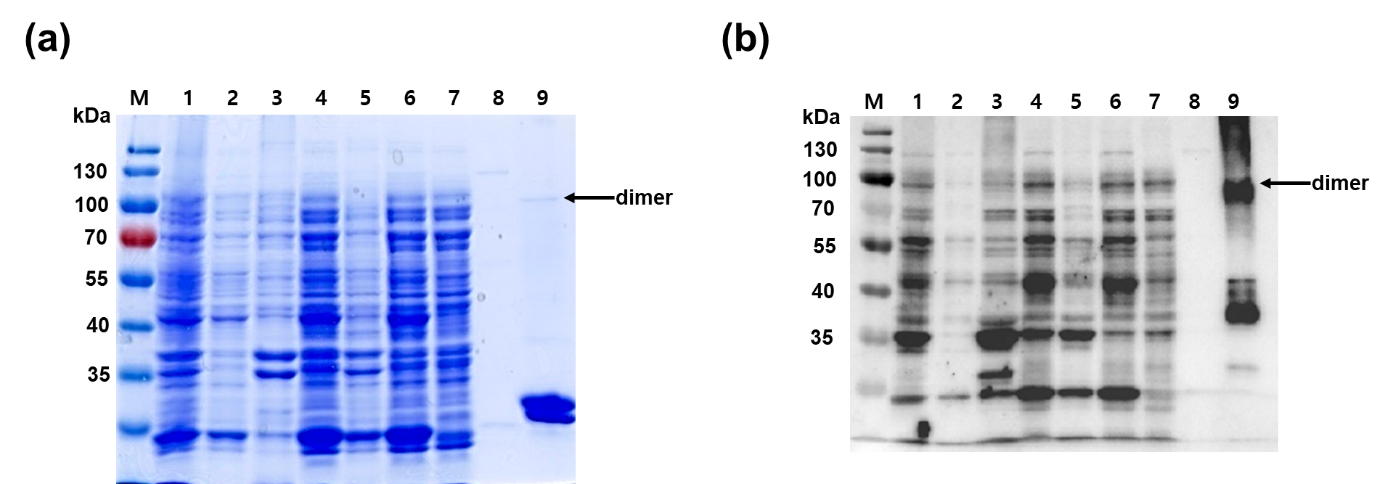


**Figure S1**. Purification of GHBR expressed from *E. coli*. (a) SDS-PAGE and (b) western blot analysis of samples obtained from each purification step. Lane M, protein size marker. Lanes 1–9 correspond to the following samples: (1) harvested cells resuspended in PBS containing 2 mM EDTA (2) soluble fraction obtained after sonication (3) insoluble fraction obtained after sonication (4) soluble fraction after solubilization (5) insoluble fraction after solubilization (6) sample after dialysis (7) flow-through fraction during FPLC injection (8) wash fraction and (9) eluted fraction.

**
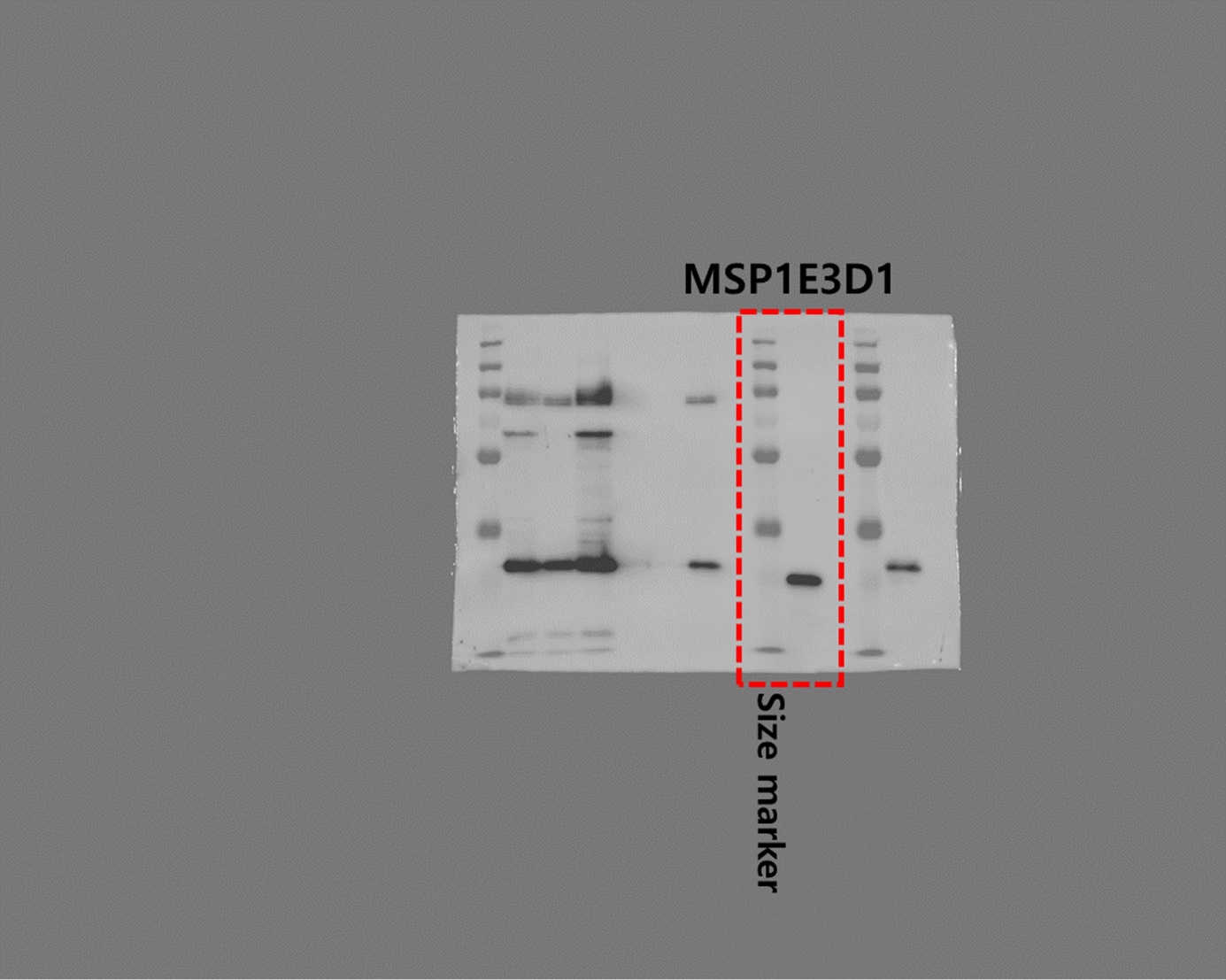
**

**Figure S2**. Uncropped original Western blot image corresponding to the blot shown in Figure 2B. The red dashed box indicates the region presented in Figure 2B.

**
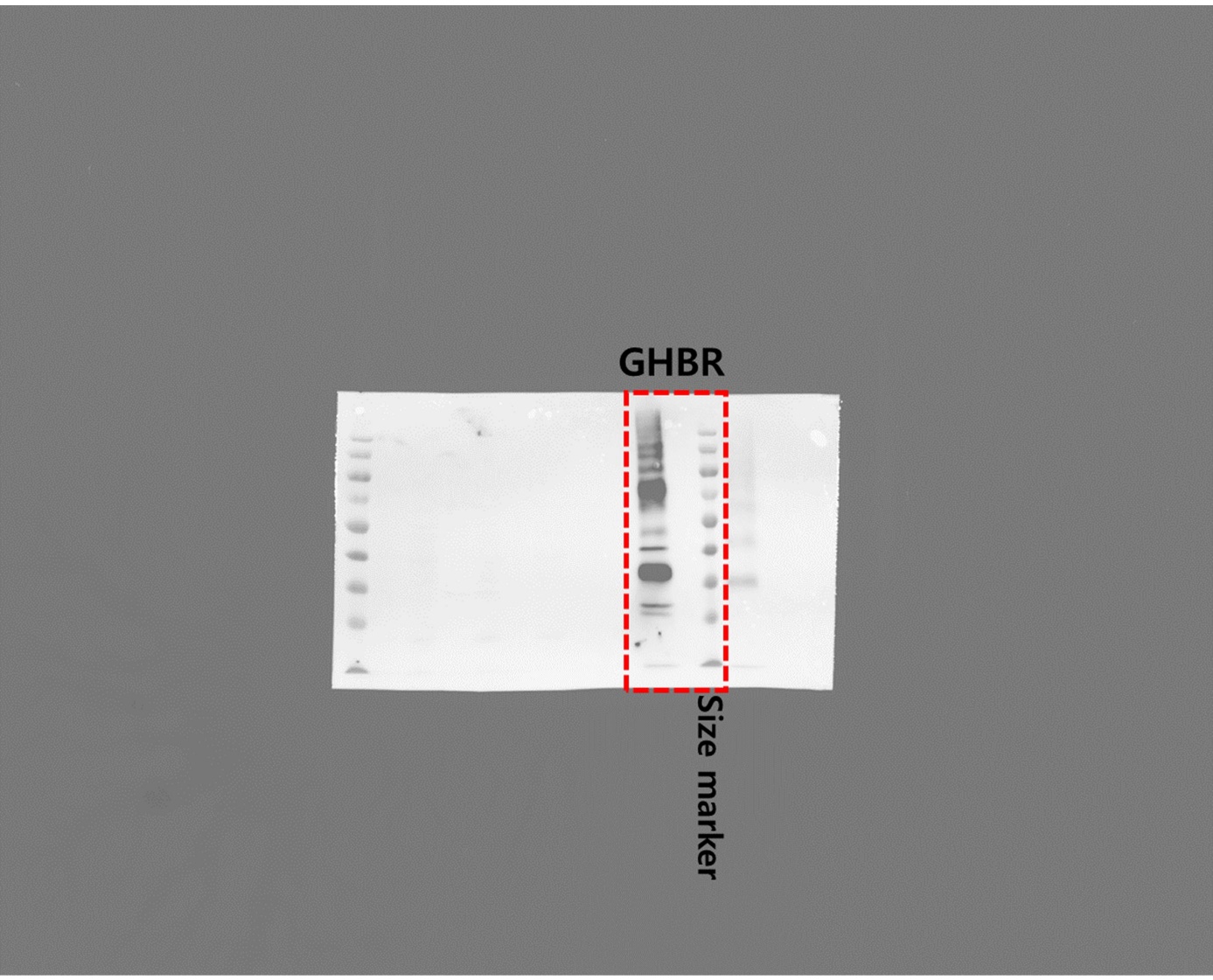
**

**Figure S3**. Uncropped original Western blot image corresponding to the blot shown in Figure 2C. The red dashed box indicates the region presented in Figure 2C.

**
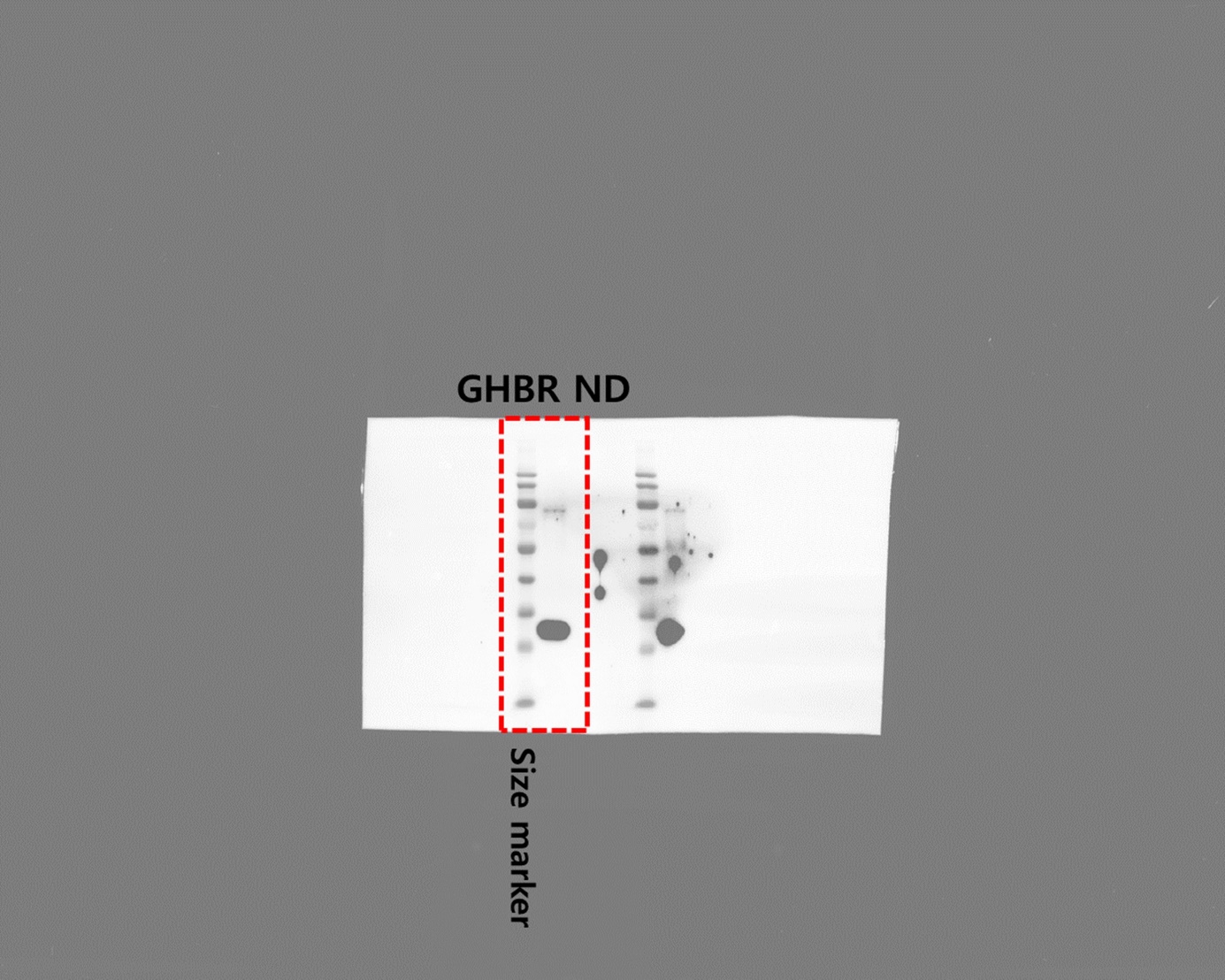
**

**Figure S4**. Uncropped original Western blot image corresponding to the blot shown in Figure 2D. The red dashed box indicates the region presented in Figure 2D.

**
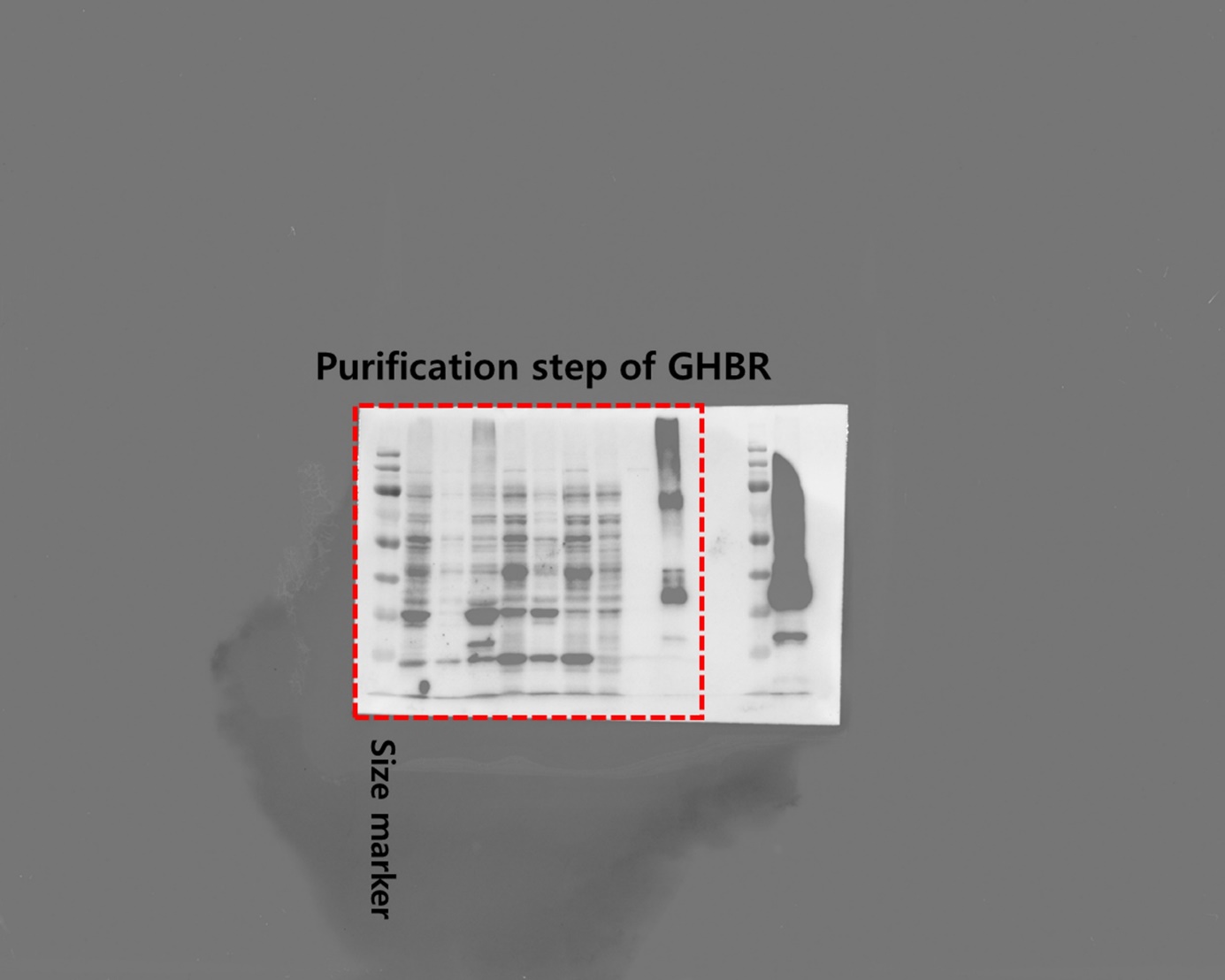
**

**Figure S5**. Uncropped original Western blot image corresponding to the blot shown in Figure S1. The red dashed box indicates the region presented in Figure S1.


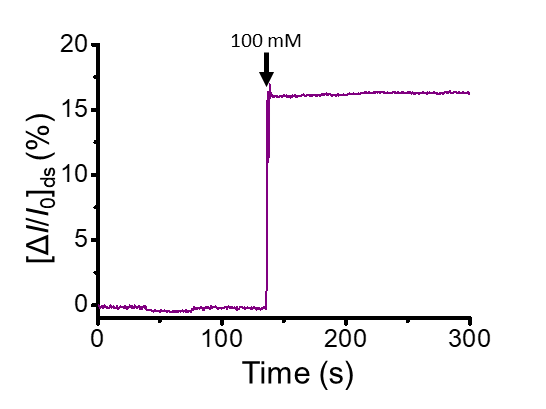


**Figure S6**. Upper limit of detection of the prestented GHB-GFET sensor.
